# Sodium Hypochlorite Pre-Oxidation as a Key Contributor to Trihalomethane Formation and Carcinogenic Risk: Evidence from Adjacent Water Treatment Plants

**DOI:** 10.3390/toxics14050361

**Published:** 2026-04-24

**Authors:** Rongjie Shi, Ning Liu, Tao Lan, Xiaoli Ye, Zhimin Peng, Li Wang, Lintao Li, Xiaoshu Yu, Chuande Chen

**Affiliations:** 1 Shenzhen Luohu District Center for Disease Control and Prevention, Shenzhen 518024, China; rosesrj0807@163.com (R.S.); shinexix1@163.com (X.Y.); fatpengpeng@163.com (Z.P.); 15811805564@163.com (L.W.); 13714325867@163.com (L.L.); 2 Shenzhen Center for Disease Control and Prevention, Shenzhen 518055, China; 13501580582@139.com (N.L.); 13501580321@163.com (T.L.); yu_xiaoshu@163.com (X.Y.)

**Keywords:** pre-oxidation, drinking water, disinfection by-product, carcinogenic risk assessment, non-carcinogenic risk

## Abstract

An on-site investigation was conducted to analyze the causes of excessive trihalomethane (THM) formation in Plant A and to mitigate the health risks associated with THM exposure in drinking water. Adjacent Plant B was used as a reference plant. Both water treatment plants used the same source water but employed different pre-oxidants. Systematic stage-specific sampling and analysis of historical monitoring data were conducted to identify the key contributing stage for THM formation. The investigation revealed that 85% of the trichloromethane in Plant A’s finished water originated from the sodium hypochlorite pre-oxidation step, identifying this stage as the key contributing stage. THM concentrations were generally higher at Plant A than at Plant B. A multi-pathway health risk assessment of THM in drinking water indicates that non-carcinogenic risks are negligible, but lifetime carcinogenic risks warrant attention. The findings demonstrate that pre-oxidant selection is a significant governing factor of finished water disinfection by-product (DBP) levels. Following these findings, Plant A implemented measures, including enhanced chlorination management, reduced pre-chlorination, and upgraded sedimentation and V-shaped filters, which substantially reduced chlorinated DBPs in the finished water.

## 1. Introduction

Waterborne diseases remain a significant global public health challenge, disproportionately affecting young children. In 2019, water pollution was responsible for an estimated 1.4 million deaths worldwide, including over 140,000 deaths among children under age five [1,2]. To ensure microbiological safety, disinfectants such as chlorine, chloramines, chlorine dioxide, and ozone are indispensable in water treatment [3]. However, these disinfectants react with natural organic matter (NOM) in water to generate disinfection by-products (DBPs, approximately 600 to 700 compounds) in the finished water, with the most common being trihalomethanes (THMs), haloacetic acids (HAAs), chlorate and chlorite, etc. [4,5]. Many DBPs, including several THMs, are of public health concern due to their cytotoxic, genotoxic, and carcinogenic properties [4,5,6]. Consequently, regulatory bodies worldwide, including those in China, have established strict limits on DBPs in drinking water (see Table 1) [7,8,9]. Multiple factors, including disinfectant choice, raw water quality parameters, and treatment processes, influence DBP formation, and these variables can act independently [5,10]. Pre-oxidation is an effective method of enhancing coagulation-sedimentation and removing algae. However, the oxidation of algal organic matter released during this process can increase DBP formation. Studies compared the effects of different pre-oxidants on THM formation. They found that pre-oxidation can affect THM levels in water: pre-chlorination increases THM concentrations, whereas pre-oxidation with ozone or chlorine dioxide can reduce THM formation [11,12,13]. Despite these insights, existing studies have primarily focused on the effect of pre-oxidants on final DBP concentrations, with limited investigation into the dynamic formation of specific DBPs across sequential treatment stages. Such field-based investigations are critical for pinpointing key contributing stages within the water treatment process. Although controlled bench-scale experiments can provide valuable mechanistic understanding, they may not fully capture the operational complexity of real-world water treatment plants, including variable source water quality, hydraulic fluctuations, and the dynamic interactions between pre-oxidation and subsequent treatment processes (e.g., coagulation, sedimentation, filtration, and disinfection).

This study was motivated by a specific public health concern: routine monitoring in a major southern Chinese city revealed that tap water from “Plant A” exceeded the national standard (GB 5749-2022) for trichloromethane (TCM) [7]. TCM, classified as a Group 2B carcinogen (possibly carcinogenic to humans) by the International Agency for Research on Cancer (IARC), has been linked to cancers of the bladder, kidney, breast, and liver [4,14,15]. To address this exceedance, we designed a field investigation to answer three distinct research questions: (1) Does the choice of pre-oxidant influence the final DBP profile in finished water under real-world operating conditions? (2) Can stage-specific field sampling accurately identify where most DBP formation occurs across sequential treatment stages? (3) Is the available evidence sufficient to guide targeted process interventions that reduce DBP levels while maintaining disinfection efficacy?

To answer these questions, we conducted a systematic, stage-by-stage analysis of Plant A’s water treatment process and employed a reference plant experiment design leveraging two adjacent plants that use identical source water but different pre-oxidation strategies. This approach allowed us to: (1) identify the key treatment stage responsible for excessive TCM formation; (2) compare the DBP profiles produced by sodium hypochlorite versus chlorine dioxide pre-oxidation, and pinpoint the key formation stage through stage-specific sampling; and (3) evaluate the effectiveness of subsequent process modifications in reducing DBPs.

## 2. Materials and Methods

### 2.1. Study Subjects

This study was conducted in a major megacity in southern China, characterized by high population density and a heavy reliance on a centralized water supply. A citywide monitoring network conducts quarterly surveillance of drinking water quality across the urban area. Two water treatment facilities were selected for the study.

**Figure 1:**
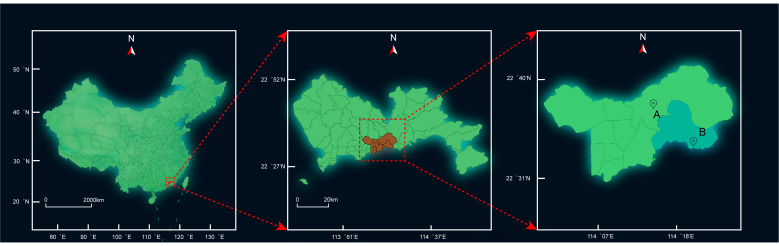
The water supply coverage of water treatment plants A and B.

**Figure 2:**
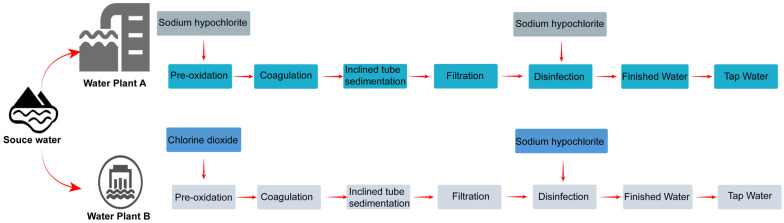
The water treatment process flows through Plants A and B.

Water Treatment Plant A, established in 1961, is the city’s oldest water treatment facility, serving a population of approximately 1.6 million. Water Treatment Plant B serves approximately 150,000 residents. Both plants draw raw water from the same reservoir and employ conventional treatment processes, including coagulation, sedimentation, filtration, and disinfection, without advanced treatment technologies. Since 2016, Plant A has used sodium hypochlorite for both pre-oxidation and disinfection, whereas Plant B employs chlorine dioxide as the pre-oxidant and sodium hypochlorite as the disinfectant. The operational parameters of both water treatment plants are shown in Table 2. Neither plant had undergone process upgrades; consequently, Plant B was selected as the reference plant. The supply areas of both plants are shown in Figure 1, and their water treatment processes are detailed in Figure 2.

### 2.2. Investigation Methods and Sample Collection

The investigation comprised process inspections, stage-specific sampling, statistical analysis of test results and historical data, and a health risk assessment. The objectives were to identify the key contributing stage for TCM formation and provide evidence-based guidance for drinking water safety.

Sample collection was conducted as follows: (1) On 11 June 2023, eight water samples were collected from key treatment stages at Plant A for preliminary analysis (Section 3.1). Rainfall had occurred within the preceding 72 h. One sample was collected at each sampling point and submitted to the municipal and district level Centers for Disease Control and Prevention (CDC) laboratories for testing to verify the consistency of the analytical results. (2) On 11 September 2023, a total of 42 samples were systematically collected from parallel treatment stages at both Plants A and B (Section 3.2). No rainfall had occurred within the preceding 72 h. Three water samples were collected at each sampling point for testing to ensure accurate results. The sampling points consisted of one each for raw water, pre-oxidation, sedimentation, and finished water, as well as three for tap water. Definitions of the sampling points are provided in Supplementary Materials Text S1. (3) Historical routine monitoring data from 2018 to 2023, comprising 272 samples from quarterly surveillance programs, were also analyzed. One sample was collected at each sampling point. It should be noted that the suite of DBPs monitored expanded in 2023, following the implementation of the national standard GB 5749-2022. TCM and chlorate were monitored consistently throughout 2018–2023, whereas BDCM, DBCM, bromoform, total THM, dichloroacetic acid, and trichloroacetic acid were added to the monitoring program in 2023.

The following routine monitoring parameters were measured and calculated in raw water, finished water, and tap water: TCM, BDCM, DBCM, bromoform, total THM, dichloroacetic acid, trichloroacetic acid, chlorate, and chlorite. Other parameters are presented in Supplementary Materials Text S2. The total THM is a risk index calculated as the sum of the ratios of each compound’s concentration to its respective guideline value. According to the formula defined in the Chinese National Standard GB 5749-2022 [7] and the WHO Guidelines for Drinking-water Quality [8], total THM is dimensionless and is calculated as follows:

$$\mathrm{Total}\;\mathrm{THM}\;=\;\frac{C_{\mathrm{TCM}}}{0.06\;\mathrm{mg}/L}\;+\;\frac{C_{\mathrm{BDCM}}}{0.06\;\mathrm{mg}/L}\;+\;\frac{C_{\mathrm{DBCM}}}{0.10\;\mathrm{mg}/L}\;+\;\frac{C_{\mathrm{Bromoform}}}{0.10\;\mathrm{mg}/L}$$

### 2.3. Quality Control and Statistical Analysis

All analyses were performed by laboratories accredited under the China Metrology Accreditation (CMA) for drinking water testing. The scope of accreditation includes all DBPs reported in this study. Field staff underwent standardized training on sampling objectives, monitoring protocols, and instrument calibration to ensure methodological consistency. Water samples were collected, preserved, and analyzed in accordance with the Standard Examination Methods for Drinking Water (GB/T 5750-2023) [16]. Fluorobenzene served as the internal standard for THM analysis, while HAAs, chlorate, and chlorite were quantified using the external standard method.

For each batch of samples, one field blank and one transport blank were included to monitor potential contamination. Field duplicates were collected at a frequency of 10% of total samples, demonstrating acceptable precision. The standard calibration curves for eight DBPs ranged from 0.00 to 0.4 mg/L, showing good linearity, with regression coefficients exceeding 0.999. Recovery rates for all analytes in spiking experiments ranged from 80.0% to 109%. A standard solution with a median concentration and a procedural blank were processed after every ten samples to evaluate the stability of responses caused by the instrument. Results were expressed as RSD, which were below 10% for all analytes. Detection limits, linear range, RSD, and other relevant information are provided in Supplementary Materials Table S1.

Given the non-normal distribution of environmental concentration data, the presence of censored data, and potentially unequal sample sizes, non-parametric methods were employed. Variables were described using medians. Descriptive comparisons across treatment stages were presented as mean ± standard deviation without inferential statistics, owing to the inherent dependence of these samples. Group comparisons between independent samples (e.g., Plants A vs. B, 2023 vs. 2024) were performed using the Mann–Whitney U test, with statistical significance set at *p* < 0.05 (two-tailed). For samples with concentrations below the detection limit, a value of half the detection limit was used as a conservative approximation. To assess the robustness of our findings, a sensitivity analysis was conducted comparing three substitution methods: (1) half the detection limit, (2) the detection limit itself, and (3) zero. Historical records and process improvement efficacy data were compiled and analyzed using SPSS 27.0 (IBM, Chicago, IL, USA).

### 2.4. Assessment of Health Risks

The four-step health risk assessment model recommended by the USEPA was adopted to evaluate the carcinogenic and non-carcinogenic risks of THMs and HAAs in drinking water from Plants A and B. The assessment considered three exposure pathways: oral, inhalation, and dermal. The framework comprised hazard identification, exposure assessment, dose–response assessment, and risk characterization [17].

Hazard Identification. According to the IARC, TCM, BDCM, bromoform, and dichloroacetic acid are classified as Group 2B (possibly carcinogenic to humans) [18,19]. According to the USEPA classification system, DBCM is categorized as Group C (not classifiable as to its carcinogenicity to humans), while trichloroacetic acid is considered to have suggestive evidence of carcinogenic potential [18,19]. DBP exposure in drinking water has been associated with increased cancer risk in some epidemiological studies; however, others have reported no significant association with colorectal cancer incidence [4,14,15,20,21].

Exposure Assessment and Dose–response Relationship. The chronic daily intake (CDI) of each DBP was calculated separately for oral (CDI_oral_, mg/(kg·day)), dermal (CDI_der_, mg/(kg·day)), and inhalation (CDI_inh_, mg/(kg·day)) [22,23,24]. Showering is a significant route for both dermal and inhalation exposure to THMs and HAAs from tap water [20,25]. Inhalation during showering contributes substantially to the overall exposure-related risk [20].

The following formulas were used for these calculations:

$${\mathrm{CDI}}_{\mathrm{oral}}\;=\;\frac{C\;\times \;\mathrm{EF}\;\times \;\mathrm{ED}\;\times \;\mathrm{IR}}{\mathrm{AT}\;\times \;\mathrm{BW}}$$

$${\mathrm{CDI}}_{\mathrm{inh}}=\frac{C_{\mathrm{air}}\;\times \;\mathrm{VR}\;\times \;\mathrm{EF}\;\times \;\mathrm{ED}\;\times \;\mathrm{ET}\;}{\mathrm{AT}\;\times \;\mathrm{BW}}$$

$${\mathrm{CDI}}_{\mathrm{der}}=\frac{C\;\times \;\mathrm{SA}\;\times \;\mathrm{PC}\;\times \;\mathrm{EF}\;\times \;\mathrm{ED}\;\times \;\mathrm{ET}}{\mathrm{AT}\;\times \;\mathrm{BW}}$$

$$C_{\mathrm{air}}=\frac{C_{0}{+C}_{t}}{2}$$

$$C_{t}=(\frac{a}{b})(1\;-{\;e}^{-\mathrm{bt}})$$

$$a=\frac{{C\;\times \;Q}_{L}\times \;(1\;-{\;e}^{-K/}Q_{L})}{V_{s}}$$

$$b=\frac{\left[\frac{Q_{L}\left(1\;-{\;e}^{-K}{/Q}_{L}\right)}{H_{i}}{+Q}_{G}\right]}{V_{s}}$$

$$H_{i}{=H}_{i-20\;{}^{\circ}C}\left[10^{-B\left(\frac{1}{T}-\frac{1}{283}\right)}\right]$$

Oral ingestion rates and body weight parameters were obtained from the Exposure Factors Handbook of the Chinese Population (Adults) for the South China population, published by the Ministry of Environmental Protection in 2014 [26]. All parameter values used in the exposure assessment are listed in Table 3 [22,25,27,28,29].

Risk Characterization. Based on toxicological data from the USEPA Integrated Risk Information System, both carcinogenic and chronic non-carcinogenic risks associated with THMs and HAAs exposure were quantitatively evaluated. The following equations were used to calculate risks via oral ingestion, dermal contact, and inhalation:

Risk_oral_ = CDI_oral_ × SF_oral_

$${\;\mathrm{Risk}}_{\mathrm{der}}=\frac{\;{\mathrm{CDI}}_{\mathrm{der}}\times {\mathrm{SF}}_{\mathrm{oral}}}{\mathrm{GIABS}}\;$$

Risk_inh_ = CDI_inh_ × SF_inh_

Risk = Risk_oral_ + Risk_der_ + Risk_inh_

$${\mathrm{Hazardous}\;\mathrm{Index}\;(\mathrm{HI})=\mathrm{HI}}_{\mathrm{oral}}+{\mathrm{HI}}_{\mathrm{der}}=\frac{{\mathrm{CDI}}_{\mathrm{oral}}}{\mathrm{RfD}}+\frac{{\mathrm{CDI}}_{\mathrm{der}}}{\mathrm{GIABS}\;\times \;\mathrm{RfD}}$$

where SF_oral_, SF_der_, and SF_inh_ respectively represent the slope factors of the oral, dermal, and inhalation routes in kg·day/mg. Gastrointestinal absorption factor (GIABS) denote the fraction of contaminant absorbed in the gastrointestinal tract (1, dimensionless), and RfD is the reference dose for chronic oral exposure (mg/kg·day). The values of these parameters for each DBP are presented in Table 4 [30,31].

Carcinogenic risk (Risk) is a dimensionless quantity typically expressed in scientific notation. According to USEPA guidelines: if the lifetime carcinogenic risk from a pollutant is <10^−6^, the risk is considered negligible; if the risk ranges between 10^−6^ and 10^−4^, it represents a potential concern and warrants attention; and if it exceeds 10^−4^, it is regarded as high and requires priority intervention.

HI is also dimensionless. HI < 1 indicates that exposure is below the threshold for adverse effects, implying no significant health concern. HI ≥ 1 suggests that the exposure dose exceeds the reference level, indicating a potential non-carcinogenic risk [32].

## 3. Results and Discussion

### 3.1. TCM Monitoring Exceedances: Initial Investigation and Hypothesis Formation

In May 2023 (wet season), routine monitoring in a major southern Chinese city revealed that tap water from Plant A exceeded the national standard (GB 5749-2022) for TCM in 10 out of 14 samples (71.43%) [7]. The TCM concentrations ranged from 0.06165 to 0.07975 mg/L, affecting multiple communities.

A preliminary field investigation was conducted at Plant A to assess its treatment processes following the detection of TCM exceedances at 10 distribution monitoring points in the second quarter of 2023. To identify the source, water samples were collected and analyzed from key treatment stages: pre-oxidation, sedimentation, post-filtration, and finished water (see Table 5). Monitoring during this phase focused solely on TCM.

The data revealed a significant trend: the concentration of TCM after the pre-oxidation stage accounted for 85.35% of that found in the finished water. This demonstrates that the pre-oxidation step using sodium hypochlorite is the primary contributor to TCM throughout the water treatment process. Furthermore, TCM concentration remained virtually unchanged between the sedimentation and post-filtration, indicating that filtration provided negligible removal of TCM. A slight increase was observed from post-filtration to finished water, consistent with additional formation during the final disinfection step. The close agreement between the two independent laboratories confirms the reproducibility of the measured TCM concentrations and rules out analytical error as a cause of the exceedance.

Based on this preliminary investigation, we hypothesized that sodium hypochlorite pre-oxidation was the primary stage in TCM generation at Plant A.

### 3.2. Identifying Key Contributing Stages via a Reference Plant Experiment

Preliminary investigations (Section 3.1) indicated that sodium hypochlorite pre-oxidation might be a significant factor contributing to TCM exceedances at Plant A. To test this hypothesis, Plant B was selected as a reference plant, as it used the same disinfectant but a different pre-oxidant. This setup enabled direct comparison of the DBP formation dynamics attributable to the pre-oxidant. Sampling and analysis were conducted concurrently across all treatment stages at both facilities. Five sampling points were selected: source water, pre-oxidation water, sedimentation water, finished water, and tap water.

The potassium permanganate index, an indicator of organic pollution and DBP formation potential, was measured in source water. Both Plants complied with the Class II surface water requirements specified in the Environmental Quality Standards for Surface Water (GB 3838-2002) [33]. The median index at Plant B (3.20 mg/L) was slightly higher than at Plant A (2.46 mg/L, *p* < 0.05), indicating a greater organic precursor load. The DBP profiles diverged markedly depending on the pre-oxidant used.

**Figure 3:**
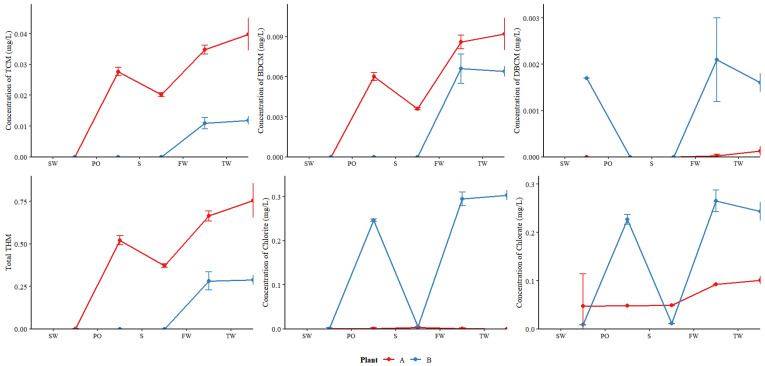
The concentrations of DBPs in water treatment processes of Plants A and B. (SW: “Source water”; PO: “Post-preoxidation”; S: “Sedimentation”; FW: “Finished water”; TW: “Tap water”. The units of TCM, BDCM, DBCM, chlorite, and chlorate are mg/L, and total THM is dimensionless.)

Water samples at all stages had a pH close to 7.0. THM concentrations were generally higher in Plant A than in Plant B, whereas chlorate and chlorite concentrations were higher in Plant B. Both plants showed substantial increases in DBP during pre-oxidation and disinfection (Figure 3).

At Plant A, TCM and BDCM in pre-oxidation water already accounted for 79.60% and 69.77%, respectively, of their finished water. At Plant B, these compounds were below the detection limit in pre-oxidation water. In tap water, TCM, BDCM, dichloroacetic acid, trichloroacetic acid and total THM were higher in Plant A than in Plant B. These results suggest that pre-oxidation is a major contributor to halogenated DBP formation and that the choice of pre-oxidant dictates DBP speciation. These findings align with laboratory studies showing that chlorine dioxide pre-oxidation reduces THM formation by rapidly reacting with organic precursors such as catechols, β-diketones, or β-keto acids [11].

At Plant A, chlorate and chlorite in pre-oxidation water accounted for 52.22% and 100.00% of finished water, respectively. At Plant B, these values were 85.35% and 83.70%. DBCM, chlorite, and chlorate concentrations were higher in Plant B than in Plant A. Thus, while chlorine dioxide for pre-oxidation suppresses THM formation, it shifts the DBP burden to chlorite and chlorate, a finding consistent with earlier studies [6,34]. This represents a clear process trade-off: reducing regulated THMs requires managing alternative by-products that also warrant monitoring.

Bromoform, dichloroacetic acid, and trichloroacetic acid were not detected at any stage of the treatment processes. All nine DBPs in finished water and tap water remained below national regulatory limits. The absence of bromoform and low detection rate of DBCM suggest low bromide levels in the source water, which originates from inland areas unaffected by seawater intrusion [11]. When the pH of water samples at each stage was close to 7, THMs formation exceeded HAAs, consistent with known pH effects [10]. Detailed DBP results at each stage are presented in Table S3, and other water quality parameters are provided in Table S2.

### 3.3. Long-Term and Seasonal Patterns of DBPs in Tap Water: Comparative Analysis of Plants A and B

In 2023, regulatory monitoring was implemented for the potassium permanganate index in source water and for DBPs, including DBCM, BDCM, bromoform, dichloroacetic acid, and trichloroacetic acid, in drinking water. Among the DBPs listed in Table 6, TCM and chlorate were consistently monitored from 2018 to 2023, enabling long-term seasonal comparisons. BDCM, DBCM, bromoform, total THM, dichloroacetic acid, and trichloroacetic acid were added to the routine monitoring program in 2023 following the updated implementation of GB 5749-2022; therefore, their analyses are based on 2023 data only.

Source water monitoring in 2023 showed that the potassium permanganate index level was slightly higher in the wet season (2.42 mg/L) than in the dry season (1.61 mg/L, *z* = −1.307, *p* > 0.05), suggesting a greater load of organic precursors during the wet season.

Analysis of routine monitoring data revealed that during the wet seasons of 2021 and 2023, two and ten water samples, respectively, from Plant A exceeded the 0.06 mg/L standard for TCM. No exceedances were observed in Plant B. Long-term monitoring data (2018–2023) for TCM and chlorate were analyzed to assess whether the seasonal and inter-plant differences observed in the 2023 stage-specific sampling were consistent over multiple years. The long-term data supported that TCM concentrations were consistently higher in Plant A than in Plant B across all years (*p* < 0.05) and that wet season TCM levels were consistently higher than dry season levels (*p* < 0.05). These findings corroborate the stage-specific observations regarding TCM formation patterns. However, long-term data for brominated THMs and HAAs were not available, as these analytes were introduced only in 2023; therefore, the stage-specific findings for those compounds are based solely on the 2023 sampling. Consistent with earlier results, tap water from Plant A showed significantly higher concentrations of TCM and BDCM, as well as a higher total THM, compared with Plant B (*p* < 0.05). In contrast, chlorate levels were elevated in Plant B [11,34,35]. These findings indicate that the choice of pre-oxidant significantly affects the DBP formation in drinking water systems [10,12]. The difference in DBCM concentration between the two plants was not statistically significant.

Seasonal analysis showed that TCM, BDCM, and total THM were consistently higher in wet season water than in dry season (*p* < 0.05). Dichloroacetic and trichloroacetic acids were detected only during the wet season. In contrast, DBCM concentrations were higher in the dry season, without statistical significance, possibly influenced by bromide levels in raw water. Chlorate concentrations showed no significant seasonal variation. The formation of chlorate showed little correlation with wet or dry season conditions. It was primarily influenced by chlorine dioxide dosage, residual levels, water temperature, pH, coagulant type, and the freshness of the sodium hypochlorite solution, consistent with earlier studies [34,36]. Detailed water quality monitoring data from 2018 to 2023 are presented in Table 6.

The detection rates of dichloroacetic acid, trichloroacetic acid, and bromoform were less than 50%, so they were not included in the comparative analysis. When TCM, bromoform, dichloroacetic acid, and trichloroacetic acid were not detected, their respective detection limits and a value of 0 were substituted into the calculations. The findings remained robust: TCM, BDCM, and total THM concentrations in drinking water were significantly higher during the wet season than during the dry season (*p* < 0.05). The relevant concentration values, as well as *z* and *p* values, are presented in Table S4.

### 3.4. Mechanistic Interpretation and Public Health Implications for DBP Control

NOM, such as humic and fulvic acids, is recognized as a major precursor of DBPs [37,38,39]. Conventional treatment processes, such as coagulation, sedimentation, and filtration, can remove a portion of these precursors, thereby ensuring disinfection efficiency while reducing the formation of chlorinated DBPs. This is particularly important during rainfall events, when controlling organic precursor levels is essential for maintaining drinking water quality [25,36,40].

In this study, both plants drew source water from the same reservoir. Although the potassium permanganate index was slightly higher at Plant B, Plant A consistently exhibited significantly higher concentrations of TCM and other chlorinated DBPs. At Plant A, the application of sodium hypochlorite during pre-oxidation initiated rapid halogenation reactions with organic matter. This step alone generated over 75% of the final TCM load, making it the key contributing stage that warrants priority intervention. In contrast, Plant B used chlorine dioxide, a stronger oxidant that primarily broke down organic precursors through oxidation rather than halogen substitution [6,10]. This not only minimized the formation of chlorinated THMs at the source but also reduced the precursor burden for the final chlorination step, resulting in substantially lower overall yield of regulated chlorinated DBPs [6,12].

The May 2023 TCM exceedance at Plant A was not an isolated anomaly but a predictable consequence of its process design under specific environmental stressors. Due to the sodium hypochlorite pre-oxidation process employed at Plant A, when the level of organic precursors surges significantly during high-flow periods, particularly during heavy rainfall, this predictable process leads to substantial increases in DBPs such as TCM in drinking water, potentially exceeding regulatory limits [3,8,14,20,23]. The widespread exceedances across Plant A’s service area in May 2023 were therefore both event-specific and, given the water treatment process design, highly likely under high-precursor conditions. The reduction to compliant levels observed during Section 3.2 may be attributed to decreased levels of organic precursors in the source water.

From a public health perspective, these findings are significant. TCM is classified as a Group 2B possible human carcinogen by the IARC, requiring application of the precautionary principle to minimize population exposure. Therefore, we propose the following improvement measures for the water treatment plant. (1) Source Control: Optimize pre-oxidant selection. Switching from free chlorine to alternative oxidants, such as chlorine dioxide or ozone, can dramatically reduce the formation of chlorinated THMs, albeit at the cost of managing new byproducts, such as chlorate, chlorite, and bromate [6,34]. (2) Process Enhancement: Strengthen the removal of organic precursors before disinfection. Enhancing coagulation, sedimentation, and filtration efficiency, especially during high-flow periods, is crucial to reduce the substrate available for DBP formation [25,38]. (3) Disinfection Optimization: Implement multi-point chlorination control and optimize pre-oxidant and disinfectant dosing. Minimizing the free chlorine dose and contact time while ensuring microbiological safety can further reduce DBP formation [31].

### 3.5. Assessment of Health Risks of THMs in Drinking Water

A multi-pathway exposure health risk assessment was conducted for those DBPs mentioned in Section 3.2. Since the detection rates of bromoform, dichloroacetic acid, and trichloroacetic acid were below 50%, only the carcinogenic and non-carcinogenic risks of TCM, BDCM, and DBCM in the tap water of Plants A and B were evaluated. The average DBP concentrations were substituted into the calculation formula. The HI for DBPs in tap water from both plants was below 1, indicating a low non-carcinogenic risk. The total carcinogenic risk attributed to TCM in tap water from Plant A exceeded 10^−4^, which is classified as high and necessitates priority intervention. In contrast, the lifetime carcinogenic risks for TCM and BDCM in tap water from Plant B, as well as for DBCM in tap water from both plants, fell within the range of 10^−6^ to 10^−4^, indicating a potential cancer risk that warrants attention. The carcinogenic risks of TCM and BDCM in tap water from Plant A were higher than those from Plant B. Notably, the carcinogenic risk of TCM in tap water from Plant B was only 29.83% of that from Plant A. Detailed carcinogenic and non-carcinogenic risk assessments are presented in Table 7 and Table 8.

Comparison of multi-pathway lifetime carcinogenic risks for the three DBPs in tap water from both plants revealed a consistent order: Risk_inh_ > Risk_oral_ > Risk_der_. The carcinogenic risks via oral ingestion and inhalation were both considerable, on the order of 10^−5^ for each route, which was several hundred times higher than that via dermal exposure. Although the multi-pathway risk assessment indicated that the oral pathway contributed a relatively large share to the total lifetime carcinogenic risk, it is important to note that boiling tap water before consumption is a deeply ingrained habit in China [37,41]. This practice effectively removes volatile THMs (e.g., TCM) through volatilization, thereby reducing the actual oral exposure risk by approximately 70% [37]. Consequently, for most residents, the real-world oral carcinogenic risk is likely to be on the order of 10^−7^, well below the commonly acceptable threshold of 10^−6^. It should be noted that the oral risk assessment presented in Table 7 and Table 8 is based on the assumption that all tap water is consumed unboiled. Nevertheless, for individuals who consume unboiled tap water, the oral pathway remains a concern and should be addressed in tailored health advisories.

With the oral pathway effectively mitigated by boiling, inhalation exposure during household activities, particularly showering and swimming, emerges as the predominant contributor to total carcinogenic risk. THMs volatilize from hot water into indoor air and are readily absorbed through the respiratory tract [12,24]. This finding has direct implications for public health guidance as modifiable lifestyle factors, such as reducing shower duration, ensuring adequate bathroom ventilation (e.g., using exhaust fans or opening windows), and avoiding very hot showers, can substantially lower inhalation risk [42]. Dermal absorption contributed the smallest fraction to the total risk across all three THMs. However, unlike the oral pathway, dermal exposure cannot be easily eliminated solely through behavioral changes, as it occurs during routine household chores, bathing, and handwashing. The consistent finding that females had slightly higher dermal risk may reflect longer time spent on household chores or more frequent handwashing [41].

Furthermore, a gender-based disparity was observed: the total multi-pathway carcinogenic risk was slightly higher for males than for females, primarily driven by their higher inhalation risk, a pattern consistent with existing literature [12,24,43]. The higher inhalation risk in males may be associated with longer, hotter showers or occupational exposures [24]; therefore, this subgroup could benefit most from advice on shower duration and ventilation. Conversely, females’ higher oral ingestion and dermal exposure may reflect different water use behaviors (e.g., more frequent dishwashing and bathing) [41]. These findings underscore the need for gender-sensitive public health messaging that acknowledges these behavioral differences and provides tailored recommendations.

### 3.6. Evaluation of Process Improvement Efficacy

As a result of the aforementioned investigation, operators of Plant A prioritized addressing the issue of excessive TCM concentrations. Despite site constraints at the existing plant and ongoing capacity expansion (including a deep-water treatment process) at the new facility, key interventions were implemented starting in October 2023. These included enhanced control of the chlorination process, reduced pre-oxidation chlorine dosing, and the commissioning of a new high-efficiency sedimentation tank coupled with a V-shaped filter.

**Figure 4:**
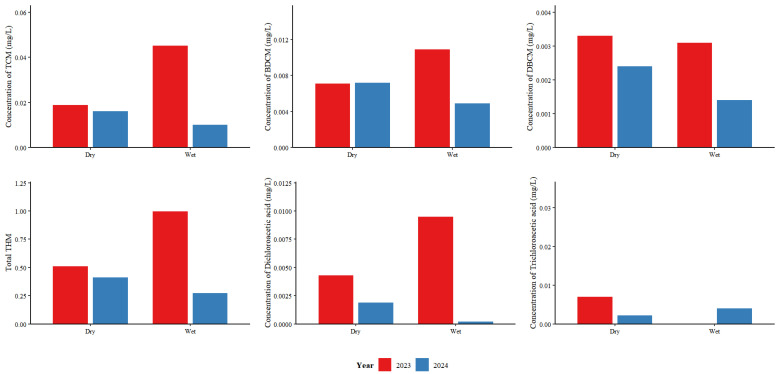
Comparison of the DBP concentrations in Plant A. (2023 vs. 2024; Dry: dry season; Wet: wet season. The units of TCM, BDCM, DBCM, dichloroacetic acid, and trichloroacetic acid are mg/L, and total THM is dimensionless. When a substance was not detected, half the detection limit was used for calculation.)

Water quality monitoring in 2024 indicated that, among THMs, TCM remained the predominant contaminant in drinking water from Plant A. During the wet season, the concentrations of TCM, DBCM, BDCM, dichloroacetic acid, and trichloroacetic acid were all significantly lower in 2024 than in 2023 (*p* < 0.05), and total THM also showed a significant decrease (*p* < 0.05). During the dry season, the concentration of DBCM in drinking water in 2024 was significantly lower than that in 2023 (*p* < 0.05). Although the concentrations of TCM, BDCM, dichloroacetic acid, trichloroacetic acid, and total THM were lower in 2024 than in 2023, these differences were not statistically significant. The reduction in DBP concentrations was more pronounced during the wet season than during the dry season. The most substantial reductions were observed for TCM (77.61%), total THM (72.59%), dichloroacetic acid (78.95%), and trichloroacetic acid (89.87%) during the wet season. The detection rate of bromoform was only 38.79%; thus, it was excluded from comparative analysis. Further details are provided in Figure 4 and Table S5.

When TCM, BDCM, DBCM, bromoform, dichloroacetic acid, and trichloroacetic acid were not detected, their respective detection limits and a value of 0 were substituted into the calculations. The results remained robust. The concentrations of TCM, DBCM, BDCM, dichloroacetic acid, and trichloroacetic acid were all significantly lower in 2024 than in 2023 (*p* < 0.05), and the total THM value also showed a significant decrease (*p* < 0.05) during the wet season. The relevant concentration values, as well as *z* and *p* values, are presented in Table S5.

## 4. Conclusions

Through an integrated analysis of stage-specific sampling, a reference plant experiment, and longitudinal monitoring data, this study consistently demonstrated that the choice of pre-oxidant was a significant influencing factor governing the speciation and concentration of DBPs in finished water. Two key scientific findings emerged. First, the sodium hypochlorite pre-oxidation stage at Plant A was the primary source of halogenated DBPs, such as TCM, and represented the key contributing stage for THM in the plant’s water supply. Second, different pre-oxidants exerted a significant influence on the formation patterns of DBPs in water supplies. Guided by these findings, targeted process optimization at Plant A achieved substantial reductions (up to 77.6%) in key DBPs, validating the efficacy of science-based interventions.

Several limitations should be acknowledged. Bromide concentrations in source water were not measured, and NOM precursors were characterized solely by the potassium permanganate index, a surrogate for overall organic carbon content. Bromide is a key precursor for the formation of brominated THMs (e.g., bromoform, DBCM). While the low detection rates of brominated THMs suggest that bromide levels in the reservoir (located inland, without seawater intrusion) are likely low, the absence of direct bromide measurements limits a more quantitative understanding of bromine incorporation pathways. Similarly, using the potassium permanganate index as the sole indicator of organic matter does not capture the specific chemical composition, functional group distribution, or reactivity of NOM fractions (e.g., humic substances, algal organic matter) that influence DBP formation and speciation. This study was conducted in a single city in southern China; therefore, the findings may not be fully generalizable to other regions with different source water characteristics, climate conditions, or treatment practices. Future studies across multiple cities and water sources would further strengthen the external validity of these conclusions.

The oral and inhalation pathways were the dominant contributors to multi-pathway carcinogenic risk, each on the order of 10^−5^, which was several hundred times higher than dermal exposure. A gender disparity was noted as males had a slightly higher total risk due to greater inhalation risk, whereas females showed higher oral and dermal risks, identifying priority groups for risk communication. 

In summary, this work bridges process engineering and public health. This study not only advances theoretical understanding of the mechanism underlying TCM formation but also offers technical pathways for precise DBP control in water treatment plants. It further provides empirical support for public health decision-making and the safe management of water resources.

## Figures and Tables

**Table 1:** DBP limits included in routine monitoring.

DBPs	China Limit (mg/L, 2022) [7]	U.S. Environmental Protection Agency (USEPA) Limit (mg/L, 2024) [9]		World Health Organization (WHO) Limit (mg/L, 2022) [8]	Disinfection Method
TCM	0.06	0.07 ^a^	/	0.30	Sodium hypochlorite, liquid Chlorine
Dibromochloromethane (DBCM)	0.10	0.06 ^a^	/	0.10	Sodium hypochlorite, liquid Chlorine
Bromodichloromethane (BDCM)	0.06	0 ^a^	/	0.06	Sodium hypochlorite, liquid Chlorine
Bromoform	0.10	0 ^a^	/	0.10	Sodium hypochlorite, liquid Chlorine
Total trihalomethane (Total THM)	1.00	/	0.08 ^b^	1.00	Sodium hypochlorite, liquid Chlorine
Dichloroacetic acid	0.05	0 ^a^	0.06 ^b,c^	0.05	Sodium hypochlorite, liquid Chlorine
Trichloroacetic acid	0.10	0.02 ^a^	0.06 ^b,c^	0.20	Sodium hypochlorite, liquid Chlorine
Bromate	0.01	/	0.01 ^b^	0.01	Ozone
Chlorite	0.70	0.80 ^a^	1.00 ^b^	0.70	Chlorine dioxide, sodium hypochlorite, liquid chlorine
Chlorate	0.70	/	/	0.70	Chlorine dioxide, sodium hypochlorite, liquid chlorine

^a^, the Maximum Contaminant Level Goals (MCLGs): the level of a contaminant in drinking water below which no known or expected health risks exist. MCLGs provide a margin of safety and are non-enforceable public health goals. ^b^, Maximum Contaminant Level (MCL): the highest permissible concentration of a contaminant in drinking water. MCLs are established as close to MCLG as possible, using the best available treatment technologies while considering cost-effectiveness; and represent enforceable standards. ^c^, MCL of HAAs. Total THM is a dimensionless risk index calculated as the sum of the ratios of each THM concentration to its respective guideline value.

**Table 2:** Operational parameters of both water treatment plants.

Water Treatment Plant	Flow Rate of the Filter Pool (m/s)	Residence/Contact Times (h)	Disinfectant Doses (mg/L)	Residual Chlorine Content of Finished Water (mg/L)	Coagulant Type	Filtration Characteristics
A	0.14	1–2	1.5–2.0	0.65–0.75	Polyaluminium Chloride	Sand-anthracite dual-media filters
B	0.28	1–2	1.3–1.4	0.65–0.75	Polyaluminium Chloride	Sand-anthracite dual-media filters

**Table 3:** Variables and parameters for the evaluation of health risks.

Parameter	Classification	Value	Unit
C (Concentration of DBPs in drinking water)		-	mg/L
C_air_ (Concentration of airborne DBPs during showering)		-	mg/m^3^
C_0_ (Initial concentration of DBPs)		0	mg/m^3^
C_t_ (Concentration of DBPs at time t)			mg/m^3^
a (Parameter)			mg/L·min
b (Parameter)			min^−1^
EF (Exposure frequency)		365	day·year^−1^
ED (Exposure duration)		83.93	year
IR (Ingestion rate of daily drinking water)	Total population	1.641	L/day
	Male	1.642	
	Female	1.656	
AT (Average time)		ED × 365	day
BW (Body weight)	Total population	59.5	kg
	Male	64.2	
	Female	55.4	
SA (Skin contact area)		1.6	m^2^
ET (Exposure Time)		13	min/d
VR (Respiratory rate)	Total population	7.9 × 10^−3^	m^3^/min
	Male	9.5 × 10^−3^	
	Female	7.5 × 10^−3^	
t (Shower time)		13	min
Q_L_ (Volumetric water flow rate)		5	L/min
K (mass transfer coefficient of a liquid phase basis)		7.4	L by each shower
Q_G_ (Volumetric flow rate)		50	L/min
V_s_ (Volume of air in the shower)		1.2	m^3^
H_i−20 °C_ (Henry’s law coefficient)	CHCl_3_	0.126	dimensionless
	CHBrCl_2_	0.076	
	CHBr_2_Cl	0.035	
	CHBr_3_	0.0175	
	ChCl_2_COOH	2.2 × 10^−7^	
	CCl_3_COOH	3.42 × 10^−7^	
T (Temperature)		313 (=40 °C)	K
B (Slope)	CHCl_3_	1830	dimensionless
	CHBrCl_2_	2130	
	CHBr_2_Cl	2273	
	CHBr_3_	2120	
	CHCl_2_COOH	3352	
	CCl_3_COOH	3634	

**Table 4:** The parameters, reference dose, slope factor, inhalation unit risk and reference concentration of DBPs.

DBPs		SF_oral_ (kg·day/mg)	SF_inh_ (kg·day/mg)	PC (cm/h)	RfD (mg/kg·day)
THMs	TCM	0.031	8.05 × 10^−2^	0.00683	0.01
	BDCM	0.062	1.3 × 10^−1^	0.00402	0.008
	DBCM	0.084	-	0.00289	0.02
	Bromoform	0.0079	3.85 × 10^−3^	0.00235	0.02
HAAs	Dichloroacetic acid	0.05	-	0.00121	0.004
	Trichloroacetic acid	0.07	-	0.00145	0.02

SF_oral_, slope factor for oral intake; SF_inh_, slope factor for inhalation; RfD, reference dose for chronic oral exposure; PC, skin permeability coefficient. These slope factors were obtained from the Integrated Risk Information System [30].

**Table 5:** Concentrations of TCM at key treatment stages of Plant A from two independent laboratories (average, mg/L).

Laboratory	Pre-Oxidation	Sedimentation	Post-Filtration	Finished Water
Municipal CDC	0.04310	0.04020	0.04050	0.05050
District CDC	0.03768	0.03805	0.03369	0.05164

Samples were collected concurrently by both laboratories from the same treatment stages. These data were obtained from a single routine monitoring measurement (*n* = 1).

**Table 6:** The concentrations of DBPs for tap water in Plants A and B (2018–2023).

DBPs		Plant A				Plant B				*z*	*p*
	Detection Rate (%)	Median	Wet Season	Dry Season	*z*	Median	Wet Season	Dry Season	*z*		
TCM	94.1	0.0339	0.0420	0.0293	−6.852 *	0.0129	0.0160	0.0095	−2.738 *	−11.334 *	<0.05
BDCM	100.0	0.0117	0.0140	0.0104	−4274 *	0.0072	0.0083	0.0063	−1.964	−3.117 *	<0.05
DBCM	100.0	0.0031	0.0030	0.0032	−1.747	0.0030	0.0025	0.0041	−1.964	−0.181	>0.05
Bromoform	12.0	0.00006	0.0004	0.00006	/	0.00006	0.00006	0.00006	/	/	/
Total THM	/	0.9300	1.3200	0.7150	−4.405 *	0.3750	0.4700	0.3000	−1.964	−2.982 *	<0.05
Dichloroacetic acid	39.5	0.0052	0.0125	0.0019	/	0.0064	0.0120	0.0019	/	/	/
Trichloroacetic acid	40.8	0.0022	0.0405	0.0022	/	0.0211	0.0460	0.0022	/	/	/
Chlorate	100.0	0.0740	0.0835	0.0680	−1.534	0.0790	0.0810	0.0785	−1.399	−1.435	>0.05

When a substance was not detected, half the detection limit was used for calculation; * stands for *p* < 0.05. These data were obtained from a single routine monitoring measurement (*n* = 1). The units for TCM, BDCM, DBCM, bromoform, dichloroacetic acid, trichloroacetic acid, and chlorate are mg/L, and total THM is dimensionless. Number of samples: TCM and chlorate (*n*_plantA_ = 168, *n*_plantB_ = 104); other DBPs (*n*_plantA_ = 50, *n*_plantB_ = 26). TCM and chlorate: long-term data (2018–2023); BDCM, DBCM, bromoform, total THM, dichloroacetic acid, trichloroacetic acid: 2023 data only (added to monitoring program in 2023).

**Table 7:** The carcinogenic and chronic non-carcinogenic risks of THMs in the tap water of Plant A.

DBPs	Variables	HI_oral_	HI_der_	HI	Risk_oral_	Risk_der_	Risk_inh_	Risk
TCM	Male	1.02 × 10^−1^	1.47 × 10^−3^	1.03 × 10^−1^	3.16 × 10^−5^	4.55 × 10^−7^	9.38 × 10^−5^	1.26 × 10^−4^
	Female	1.19 × 10^−1^	1.70 × 10^−2^	1.21 × 10^−1^	3.69 × 10^−5^	5.27 × 10^−7^	8.58 × 10^−5^	1.23 × 10^−4^
	Total population	1.10 × 10^−1^	1.58 × 10^−3^	1.11 × 10^−1^	3.40 × 10^−5^	4.91 × 10^−7^	8.42 × 10^−5^	1.19 × 10^−4^
BDCM	Male	2.94 × 10^−2^	2.50 × 10^−4^	2.97 × 10^−2^	1.46 × 10^−5^	1.24 × 10^−7^	2.11 × 10^−5^	3.59 × 10^−5^
	Female	3.44 × 10^−2^	2.89 × 10^−4^	3.47 × 10^−2^	1.71 × 10^−5^	1.44 × 10^−7^	1.49 × 10^−5^	3.21 × 10^−5^
	Total population	3.17 × 10^−2^	2.69 × 10^−4^	3.20 × 10^−2^	1.57 × 10^−5^	1.34 × 10^−7^	1.90 × 10^−5^	3.48 × 10^−5^
DBCM	Male	1.66 × 10^−4^	1.01 × 10^−6^	1.67 × 10^−4^	2.79 × 10^−7^	1.70 × 10^−9^	-	2.81 × 10^−7^
	Female	1.94 × 10^−4^	1.18 × 10^−6^	1.95 × 10^−4^	3.26 × 10^−7^	1.98 × 10^−9^	-	3.28 × 10^−7^
	Total population	1.79 × 10^−4^	1.09 × 10^−6^	1.80 × 10^−4^	3.01 × 10^−7^	1.84 × 10^−9^	-	3.03 × 10^−7^

**Table 8:** The carcinogenic and chronic non-carcinogenic risks of THMs in the tap water of Plant B.

DBPs	Variables	HI_oral_	HI_der_	HI	Risk_oral_	Risk_der_	Risk_inh_	Risk
TCM	Male	3.04 × 10^−2^	4.39 × 1.0^−4^	3.09 × 10^−2^	9.44 × 10^−6^	1.36 × 10^−7^	2.80 × 10^−5^	3.76 × 10^−5^
	Female	3.56 × 10^−2^	5.09 × 10^−4^	3.61 × 10^−2^	1.10 × 10^−5^	1.58 × 10^−7^	2.57 × 10^−5^	3.68 × 10^−5^
	Total population	3.28 × 10^−2^	4.74 × 10^−4^	3.33 × 10^−2^	1.02 × 10^−5^	1.47 × 10^−7^	2.52 × 10^−5^	3.55 × 10^−5^
BDCM	Male	2.05 × 10^−2^	1.74 × 10^−4^	2.06 × 10^−2^	1.01 × 10^−5^	8.61 × 10^−8^	1.47 × 10^−5^	2.49 × 10^−5^
	Female	2.39 × 10^−2^	2.01 × 10^−4^	2.41 × 10^−2^	1.19 × 10^−5^	9.98 × 10^−8^	1.04 × 10^−5^	2.23 × 10^−5^
	Total population	2.21 × 10^−2^	1.87 × 10^−4^	2.23 × 10^−2^	1.09 × 10^−5^	9.30 × 10^−8^	1.32 × 10^−5^	2.42 × 10^−5^
DBCM	Male	2.05 × 10^−3^	1.25 × 10^−5^	2.06 × 10^−3^	3.44 × 10^−6^	2.10 × 10^−8^	-	3.46 × 10^−6^
	Female	2.39 × 10^−3^	1.45 × 10^−5^	2.41 × 10^−3^	4.02 × 10^−6^	2.43 × 10^−8^	-	4.04 × 10^−6^
	Total population	2.21 × 10^−3^	1.35 × 10^−5^	2.22 × 10^−3^	3.71 × 10^−6^	2.26 × 10^−8^	-	3.73 × 10^−6^

## Data Availability

The data are available from the corresponding author upon reasonable request.

## Supplementary Materials

The following supporting information can be downloaded at: https://www.mdpi.com/article/10.3390/toxics14050361/s1, Table S1: The detection limit, linear range, regression coefficient, spike recovery and RSD of DBPs. Table S2: Concentrations of other indicators in each process of the two water treatment plants. Table S3: Concentrations of DBPs in each water treatment process of Plants A and B (average ± standard deviation). Table S4: The concentrations of DBPs for tap water in Plants A and B (2018–2023). Table S5: Comparison of the concentrations of DBPs in drinking water during the corresponding periods of 2023 and 2024 from Plant A. Text S1: Definitions of the sampling points. Text S2: Investigation methods.

## Abbreviations

The following abbreviations are used in this manuscript:

BDCM	Bromodichloromethane
CDC	Center for Disease Control and Prevention
CDI	Chronic Daily Intake
CMA	China Metrology Accreditation
DBCM	Dibromochloromethane
DBP	Disinfection By-product
GIABS	Gastrointestinal Absorption Factor
HAA	Haloacetic acid
HI	Hazardous Index
IRIS	Integrated Risk Information System
IARC	International Agency for Research on Cancer
MCL	Maximum Contaminant Level
MCLG	Maximum Contaminant Level Goal
NOM	Natural organic matter
RAIS	Regulatory Authority Information System
SF	Slope Factor
TCM	Trichloromethane
Total THM	Total Trihalomethane
THM	Trihalomethane
USEPA	U.S. Environmental Protection Agency
WHO	World Health Organization

## References

[1] Pruss-Ustun A., Wolf J., Bartram J., Clasen T., Cumming O., Freeman M.C., Gordon B., Hunter P.R., Medlicott K., Johnston R. (2019). Burden of disease from inadequate water, sanitation and hygiene for selected adverse health outcomes: An updated analysis with a focus on low- and middle-income countries. Int. J. Hyg. Environ. Health.

[2] World Health Organization (WHO) The Global Health Observatory Water, Sanitation and Hygiene: Burden of Disease. https://www.who.int/data/gho/data/.

[3] Mian H.R., Hu G., Hewage K., Rodriguez M.J., Sadiq R. (2018). Prioritization of unregulated disinfection by-products in drinking water distribution systems for human health risk mitigation: A critical review. Water Res..

[4] Helte E., Soderlund F., Save-Soderbergh M., Larsson S.C., Akesson A. (2025). Exposure to Drinking Water Trihalomethanes and Risk of Cancer: A Systematic Review of the Epidemiologic Evidence and Dose-Response Meta-Analysis. Environ. Health Perspect..

[5] Srivastav A.L., Patel N., Chaudhary V.K. (2020). Disinfection by-products in drinking water: Occurrence, toxicity and abatement. Environ. Pollut..

[6] Sorlini S., Gialdini F., Biasibetti M., Collivignarelli C. (2014). Influence of drinking water treatments on chlorine dioxide consumption and chlorite/chlorate formation. Water Res..

[7] (2022). Standards for Drinking Water Quality.

[8] World Health Organization (WHO) Guidelines for Drinking-Water Quality: Fourth Edition Incorporating the First and Second Addenda. https://www.who.int/publications/i/item/9789240045064.

[9] United States Environmental Protection Agency (US EPA) National Primary Drinking Water Regulations. https://www.epa.gov/ground-water-and-drinking-water/national-primary-drinking-water-regulations.

[10] Kim J., Chung Y., Shin D., Kim M., Lee Y., Lim Y., Lee D. (2003). Chlorination by-products in surface water treatment process. Desalination.

[11] Gallard H., von G.U. (2002). Chlorination of natural organic matter: Kinetics of chlorination and of THM formation. Water Res..

[12] Sheng D., Bu L., Zhu S., Wu Y., Wang J., Li N., Zhou S. (2022). Impact of pre-oxidation on the formation of byproducts in algae-laden water disinfection: Insights from fluorescent and molecular weight. J. Environ. Sci..

[13] Teksoy A., Alkan U., Başkaya H.S. (2008). Influence of the treatment process combinations on the formation of THM species in water. Sep. Purif. Technol..

[14] Semerjian L., Dennis J. (2007). Multipathway risk assessment of trihalomethane exposure in drinking water of Lebanon. J. Water Health.

[15] Villanueva C.M., Gracia-Lavedan E., Bosetti C., Righi E., Molina A.J., Martin V., Boldo E., Aragones N., Perez-Gomez B., Pollan M. (2017). Colorectal Cancer and Long-Term Exposure to Trihalomethanes in Drinking Water: A Multicenter Case-Control Study in Spain and Italy. Env. Health Perspect..

[16] (2023). Standard Examination Methods for Drinking Water.

[17] Wang L., Li J., Zheng J., Liang J., Li R., Gong Z. (2022). Source tracing and health risk assessment of phthalate esters in household tap-water: A case study of the urban area of Quanzhou, Southeast China. Ecotoxicol. Environ. Saf..

[18] International Agency for Research on Cancer (IARC) List of Classifications—IARC Monographs on the Identification of Carcinogenic Hazards to Humans. https://monographs.iarc.who.int/list-of-classifications.

[19] Richardson S.D., Plewa M.J., Wagner E.D., Schoeny R., Demarini D.M. (2007). Occurrence, genotoxicity, and carcinogenicity of regulated and emerging disinfection by-products in drinking water: A review and roadmap for research. Mutat. Res..

[20] Wang G.S., Deng Y.C., Lin T.F. (2007). Cancer risk assessment from trihalomethanes in drinking water. Sci. Total Environ..

[21] Mendy A. (2025). Disinfection byproducts in US drinking water and cancer mortality. Int. J. Environ. Health Res..

[22] Amjad H., Hashmi I., Rehman M.S., Ali Awan M., Ghaffar S., Khan Z. (2013). Cancer and non-cancer risk assessment of trihalomethanes in urban drinking water supplies of Pakistan. Ecotoxicol. Environ. Saf..

[23] Lee S.C., Guo H., Lam S.M., Lau S.L. (2004). Multipathway risk assessment on disinfection by-products of drinking water in Hong Kong. Environ. Res..

[24] Siddique A., Saied S., Mumtaz M., Hussain M.M., Khwaja H.A. (2015). Multipathways human health risk assessment of trihalomethane exposure through drinking water. Ecotoxicol. Environ. Saf..

[25] Pardakhti A.R., Bidhendi G.R., Torabian A., Karbassi A., Yunesian M. (2011). Comparative cancer risk assessment of THMs in drinking water from well water sources and surface water sources. Environ. Monit. Assess..

[26] Ministry of Ecology and Environment of the People’s Republic of China (2013). Manual of Exposure Parameters for Chinese Population.

[27] Li J., Chen J., Hu Z., Li X., Li M., Wang Y., Zhang Z., Liang X. (2023). Overlooked inorganic DBPs in trichloroisocyanuric acid (TCCA) disinfected indoor swimming pool: Evidences from concentration, cytotoxicity, and human health risk. Chemosphere.

[28] Viana R.B., Cavalcante R.M., Braga F.M., Viana A.B., de Araujo J.C., Nascimento R.F., Pimentel A.S. (2009). Risk assessment of trihalomethanes from tap water in Fortaleza, Brazil. Environ. Monit. Assess..

[29] Public Hygiene and Health Commision of Shenzhen Municipality Shenzhen Residents’ Health White Paper. https://wjw.sz.gov.cn/ztzl/szsjmjkbps2022/.

[30] Integrated Risk Information System (IRIS). List of Substances on IRIS. https://iris.epa.gov/AtoZ/?list_type=alpha.

[31] Regulatory Authority Information System (RAIS). Toxicity Values and Physical Paramters Search. https://rais.ornl.gov/cgi-bin/tools/TOX_search?select=chemtox.

[32] Li T., Du Y., Ban J., Wang Q., Sun Q. (2022). Environmental Health Risk Research: Methods and Applications.

[33] (2002). Environmental Quality Standards for Surface Water.

[34] Al-Otoum F., Al-Ghouti M.A., Ahmed T.A., Abu-Dieyeh M., Ali M. (2016). Disinfection by-products of chlorine dioxide (chlorite, chlorate, and trihalomethanes): Occurrence in drinking water in Qatar. Chemosphere.

[35] Yang X., Guo W., Lee W. (2013). Formation of disinfection byproducts upon chlorine dioxide preoxidation followed by chlorination or chloramination of natural organic matter. Chemosphere.

[36] World Health Organization (WHO) Chlorine Dioxide, Chlorite and Chlorate in Drinking-Water. https://www.who.int/docs/default-source/wash-documents/wash-chemicals/chlorine-dioxide-chlorite-chlorate-background-document.pdf.

[37] Liang X., Qian G., Wang Y., Chen M., Liu Y., Zhao P., Li J., Wang Y., Liu Y. (2024). Annual and Seasonal Variability of Trichloromethane in Drinking Water of Kunshan City 2016–2022 and Associated Health Risks. Toxics.

[38] Xiao R., Deng Y., Xu Z., Chu W. (2024). Disinfection byproducts and their precursors in drinking water sources: Origins, influencing factors, and environmental insights. Engineering.

[39] Bond T., Goslan E.H., Parsons S.A., Jefferson B. (2012). A critical review of trihalomethane and haloacetic acid formation from natural organic matter surrogates. Environ. Technol. Rev..

[40] Delpla I., Rodriguez M.J. (2016). Experimental disinfection by-product formation potential following rainfall events. Water Res..

[41] Wang Y., Zhu G., Engel B. (2019). Health risk assessment of trihalomethanes in water treatment plants in Jiangsu Province, China. Ecotoxicol. Environ. Saf..

[42] Zheng X., Xu J., Gao Y., Li W., Chen Y., Geng H., Yue J., Xu M. (2023). Within-day variation and health risk assessment of trihalomethanes (THMs) in a chlorinated indoor swimming pool in China. Environ. Sci. Pollut. Res. Int..

[43] Wang L., Fang Z., Zhou X., Cheng K., Ren Y., Li C., Gao B., Lv Y., Xu S., Xu H. (2025). Health risk assessment via ingestion of disinfection by-products in drinking water. Sci. Rep..

