# Supplementary material for: Sodium Hypochlorite Pre-Oxidation as a Key Contributor to Trihalomethane Formation and Carcinogenic Risk: Evidence from Adjacent Water Treatment Plants
Source: Toxics. 2026 Apr 24;14(5):361. doi: 10.3390/toxics14050361 (PMC13211151; doi:10.3390/toxics14050361)
Supplement: Supplementary file 1 [file toxics-14-00361-s001.zip › toxics-4264755-supplementary.pdf]

Supplementary Materials

Text S1: Definitions of the sampling points
Text S2: Investigation methods
Table S1. The detection limit, linear range, regression coefficient, spike recovery and RSD of DBPs.
Table S2. Concentrations of other indicators in each process of the two water treatment plants.
Table S3. Concentrations of DBPs in each water treatment process of Plants A and B (average ± standard deviation).
Table S4. The concentrations of DBPs for tap water in Plants A and B (2018-2023).
Table S5. Comparison of the concentrations of DBPs in drinking water during the corresponding periods of 2023 and 2024 from Plant A.

Text S1: Definitions of the sampling points

Source water: Raw water inlet before any treatment. Pre-oxidation water: Immediately after the pre-oxidation tank or basin (after chemical addition and mixing). Sedimentation water: Outlet of the inclined tube sedimentation tank. Post-filtration water: Outlet of the sand filtration unit, after filtration but before disinfection. Finished water: After completion of all treatment processes (including disinfection) and before entering the distribution system. Tap water: Collected from household taps in the distribution system, after flushing for 5 minutes to ensure representative samples.

Text S2: Investigation methods

For raw water, the following parameters were measured: pH, potassium permanganate index (expressed as O_2), ammonia (expressed as N), trichloromethane (TCM), bromodichloromethane (BDCM), dibromochloromethane (DBCM), bromoform, total trihalomethane (total THM), dichloroacetic acid, trichloroacetic acid, chlorate, chlorite, total coliforms, and total bacterial count.

For finished and tap water, the following parameters were assessed: pH, potassium permanganate index (expressed as O_2), ammonia (expressed as N), TCM, BDCM, DBCM, bromoform, total THM, dichloroacetic acid, trichloroacetic acid, chlorate, chlorite, total coliforms, total bacterial count.

Table S1. The detection limit, linear range, regression coefficient, spike recovery rate and RSD of DBPs.

Index	Detection limit (mg/L)	Linear range (mg/L)	Regression coefficient (R^2)	Spike recovery rate (%)	RSD (measurement uncertainty, %)
TCM	0.00003	0-0.054	0.9999	88.1-99.6	4.7
BDCM	0.00008	0-0.054	0.9996	96.2-102.0	2.6
DBCM	0.00004	0-0.055	0.9996	94.8-105.0	2.7
Bromoform	0.00012	0-0.054	0.9993	95.5-103.0	3.1
Chlorite	0.0001	0-0.4	0.9999	93.2-107.0	6.1
Chlorate	0.0005	0-0.4	0.9999	83.9-109.0	5.1
Dichloroacetic acid	0.0037	0-0.2	0.9999	80.0-102.0	1.1
Trichloroacetic acid	0.0044	0-0.2	0.9999	80.0-102.0	6.9

Table S2. Concentrations of other indicators in each process of the two water treatment plants.

Index	Plant A					Plant B				
	Source water	Pre-oxidation	Sedimentation	Finished water	Tap water	Source water	Pre-oxidation	Sedimentation	Finished water	Tap water
pH	6.93	6.77	6.79	6.79	6.83	6.81	6.78	6.72	6.70	6.86
Ammonia (expressed as N)	0.017	0.001	0.001	0.001	0.001	0.023	0.001	0.001	0.001	0.001
Potassium permanganate index (expressed as O_2)	2.461	1.904	0.736	0.491	0.778	3.197	1.093	1.032	0.621	0.640
Total bacterial count	59133.3	71	6.67	ND	ND	5600	1.67	14933.33	ND	0.56
Total coliforms	>200.5	ND	ND	ND	ND	>200.5	ND	>200.5	ND	ND

ND stands for not detected. The units of ammonia (expressed as N) and potassium permanganate index (expressed as O_2) are mg/L; total bacterial count and total coliforms are CFU/100mL; pH is dimensionless.

Table S3. Concentrations of DBPs in each water treatment process of Plants A and B (average ± standard deviation).

Index	Plant A					Plant B				
	**Source water**	**Pre-oxidation**	**Sedimentation**	**Finished water**	**Tap water**	**Source water**	**Pre-oxidation**	**Sedimentation**	**Finished water**	**Tap water**
TCM	ND	0.0277±0.0014	0.0202±0.0006	0.0348±0.0014	0.0398±0.0053	ND	ND	ND	0.0109±0.0006	0.0119±0.0006
BDCM	ND	0.0060±0.0003	0.0036±0.0001	0.0086±0.0005	0.0092±0.0012	ND	ND	ND	0.0066±0.0004	0.0064±0.0004
DBCM	ND	ND	ND	ND	0.00013±0.00004	ND	ND	ND	0.0021±0.0009	0.0016±0.0002
Total THM	ND	0.5220±0.0270	0.3730±0.0100	0.6650±0.0290	0.7570±0.0101	ND	ND	ND	0.2830±0.0140	0.2890±0.0280
Bromoform	ND	ND	ND	ND	ND	ND	ND	ND	ND	ND
Chlorite	0.0012±0.0021	0.0013±0.0022	0.0038±0.0001	0.0011±0.0019	0.0001±0.0003	0.0012±0.0021	0.2465±0.0030	0.0056±0.0001	0.2945±0.0155	0.3036±0.0117
Chlorate	0.0477±0.0670	0.0482±0.00003	0.0488±0.0005	0.0923±0.0001	0.1011±0.0078	0.0088±0.0005	0.2267±0.0099	0.0115±0.0003	0.2656±0.0225	0.2440±0.0192
Dichloroacetic acid	ND	0.0110±0.0003	ND	ND	0.0090±0.0034	ND	ND	ND	ND	ND
Trichloroacetic acid	ND	ND	ND	ND	0.0096±0.0037	ND	ND	ND	ND	ND

All samples were collected in triplicate (n = 3) at each sampling point. ND stands for not detected. When a substance was not detected, half the detection limit was used for calculation. The units of TCM, BDCM, DBCM, dichloroacetic acid, trichloroacetic acid, chlorite, chlorate and bromoform are mg/L, and total THM is dimensionless.

Table S4. The concentrations of DBPs for tap water in Plants A and B (2018-2023).

DBPs	Detection rate (%)	Plant A				Plant B				*z*	*P*
		Median	Wet season	Dry season	*z*	Median	Wet season	Dry season	*z*		
The detection limit was used for calculation											
TCM	94.1	0.0339	0.0420	0.0292	-6.852*	0.0129	0.0160	0.0095	-2.738*	-11.334*	<0.05
Total THM	/	0.9300	1.3200	0.7150	-4.405*	0.3750	0.4700	0.3000	-1.964	-2.982*	<0.05
Bromoform	12.0	0.0001	0.0001	0.0004	/	0.0001	0.0001	0.0001	/	/	/
Dichloroacetic acid	39.5	0.0062	0.0125	0.0037	/	0.0074	0.0120	0.0037	/	/	/
Trichloroacetic acid	40.8	0.0044	0.0405	0.0044	/	0.0222	0.0460	0.0044	/	/	/
Zero was used for calculation											
TCM	94.1	0.0339	0.0420	0.0292	-6.852*	0.0129	0.0160	0.0095	-2.738*	-11.334*	<0.05
Total THM	/	0.9300	1.3200	0.7150	-4.405*	0.3750	0.4700	0.3000	-1.964	-2.982*	<0.05
Bromoform	12.0	0.0000	0.0000	0.0003	/	0.0000	0.0000	0.0000	/	/	/
Dichloroacetic acid	39.5	0.0043	0.0125	0.0000	/	0.0055	0.0120	0.0000	/	/	/
Trichloroacetic acid	40.8	0.0000	0.0405	0.0000	/	0.0200	0.0460	0.0000	/	/	/

When a substance was not detected, the detection limit and zero were used for calculation; * stands for $p < 0.05$. These data were obtained from single routine monitoring measurement (n = 1). The units of TCM, BDCM, DBCM, bromoform, trichloroacetic acid and dichloroacetic acid are mg/L; total THM is dimensionless. Number of samples: TCM and chlorate ($n_{plantA} = 168$, $n_{plantB} = 104$); other DBPs ($n_{plantA} = 50$, $n_{plantB} = 26$). TCM and chlorate: long-term data (2018-2023); BDCM, DBCM, bromoform, total THM, dichloroacetic acid, trichloroacetic acid: 2023 data only (added to monitoring program in 2023).

Table S5. Comparison of the concentrations of DBPs in drinking water during the corresponding periods of 2023 and 2024 from Plant A.

DBPs	Detection rate (%)	Dry season (n = 60)				Wet season (n = 78)			
		2023	2024	*z*	*P*	2023	2024	*z*	*P*
Half the detection limit was used for calculation									
TCM	99.3	0.0189	0.0161	-0.685	>0.05	0.0451	0.0101	-7.314*	<0.05
BDCM	100.0	0.0071	0.0072	-0.226	>0.05	0.0109	0.0049	-6.443*	<0.05
DBCM	100.0	0.0033	0.0024	-6.039*	<0.05	0.0031	0.0014	-6.196*	<0.05
Bromoform	38.8	0.0004	0.0001	/	/	0.00006	0.0002	/	/
Total THM	/	0.5100	0.4118	-1.152	>0.05	0.9950	0.2727	-6.380*	<0.05
Dichloroacetic acid	50.7	0.0043	0.0019	-0.754	>0.05	0.0095	0.0020	-4.457*	<0.05
Trichloroacetic acid	51.4	0.0070	0.0022	-0.239	>0.05	0.0395	0.0040	-2.106*	<0.05
The detection limit was used for calculation									
TCM	99.3	0.0189	0.0161	-0.685	>0.05	0.0451	0.0101	-7.314*	<0.05
BDCM	100.0	0.0071	0.0072	-0.226	>0.05	0.0109	0.0049	-6.443*	<0.05
DBCM	100.0	0.0033	0.0024	-6.039*	<0.05	0.0031	0.0014	-6.196*	<0.05
Bromoform	38.8	0.0004	0.0001	/	/	0.0001	0.0003	/	/
Total THM	/	0.5100	0.4118	-1.152	>0.05	0.9950	0.2727	-6.380*	<0.05
Dichloroacetic acid	50.7	0.0043	0.0037	-1.949	>0.05	0.0095	0.0037	-2.807*	<0.05
Trichloroacetic acid	51.4	0.0070	0.0044	-0.239	>0.05	0.0395	0.0051	-2.106*	<0.05
Zero was used for calculation									
TCM	99.3	0.0189	0.0161	-0.685	>0.05	0.0451	0.0101	-7.314*	<0.05
BDCM	100.0	0.0071	0.0072	-0.226	>0.05	0.0109	0.0049	-6.443*	<0.05
DBCM	100.0	0.0033	0.0024	-6.039*	<0.05	0.0031	0.0014	-6.196*	<0.05
Bromoform	38.8	0.0003	0.0001	/	/	0.0000	0.0002	/	/
Total THM	/	0.5100	0.4118	-1.152	>0.05	0.9950	0.2727	-6.380*	<0.05
Dichloroacetic acid	50.7	0.0043	0.0000	-0.428	>0.05	0.0095	0.0000	-2.807*	<0.05
Trichloroacetic acid	51.4	0.0070	0.0000	-0.239	>0.05	0.0395	0.0029	-2.106*	<0.05

When a substance was not detected, half the detection limit, the detection limit and zero were used for calculation. These data were obtained from a single routine monitoring measurement (n = 1). The units of TCM, BDCM, DBCM, dichloroacetic acid, trichloroacetic acid, and bromoform are mg/L, and total THM is dimensionless.
